# Draft Genome Sequence of a Methanogenic Archaeon from West Spitsbergen Permafrost

**DOI:** 10.1128/mra.00938-21

**Published:** 2022-02-10

**Authors:** Vladimir Trubitsyn, Elizaveta Rivkina, Viktoria Shcherbakova

**Affiliations:** a Skryabin Institute of Biochemistry and Physiology of Microorganisms, Pushchino Scientific Center for Biological Research of the Russian Academy of Sciences, Pushchino, Moscow Oblast, Russia; b Institute of Physicochemical and Biological Problems in Soil Sciences, Pushchino Scientific Center for Biological Research of the Russian Academy of Sciences, Pushchino, Moscow Oblast, Russia; Portland State University

## Abstract

*Methanobacterium* sp. strain VT is a psychrotolerant methanogenic archaeon that was isolated from West Spitsbergen island (Norway) permafrost. This article describes the draft genome sequence of *Methanobacterium* sp. strain VT.

## ANNOUNCEMENT

The strictly anaerobic, psychrotolerant, nonmotile, hydrogenotrophic, rod-shaped, methanogenic archaeon strain VT was isolated from a West Spitsbergen permafrost sample that was obtained near Barentsburg, Svalbard archipelago, Norway (78.03N, 14.323611E) (1). The sample was delivered to the study site in a frozen state. Strain isolation was performed according to the Hungate anaerobic method (2) with antibiotics. Strain VT was grown on DSMZ 141 medium (3) with H_2_ plus CO_2_ and formate as the substrates, at a temperature of 20°С, pH of 6.6, and NaCl concentration of 0.075 M.

Phylogenetic analysis of 16S rRNA gene nucleotide sequences using the gBlocks (4) and MEGA X (5) programs showed that the strain clustered with representatives of the *Methanobacterium* genus. The closest type strain was Methanobacterium lacus 17A1 (6), with a similarity of 97.02%.

Genomic DNA preparation and sequencing were performed by the BioSpark Company (Troitsk, Russia). Genomic DNA was isolated with the FastDNA spin kit (MP Biomedicals, USA) by the column method with deposition on silica gel. The libraries were synthesized using KAPA HyperPlus kits (Kapa Biosystems, USA) in accordance with the manufacturer’s recommendations. Sequencing was performed on the Illumina NovaSeq 6000 platform, and a paired-end library with a total of 21,432,200 reads and a read length of 101 bp was obtained.

The quality of the reads was controlled with FastQC v. 0.11.5 (7). The reads were edited and filtered using Trimmomatic v. 0.36 (8) with adapter clipping (adapter library TruSeq3-PE-2).

Analysis with Kaiju v. 1.7.3 (9) showed that the read libraries contained genetic material of other organisms. Draft assembly was determined with metaSPAdes v. 3.13.0 (10), with subsequent contig binning with CONCOCT v. 1.1 (11). Alignment with Bowtie2 v. 2.3.2 (12) demonstrated that the VT genome bin accounted for 87.38% of the reads, with a coverage of 709.51× ± 147.94×. Genome completeness, as assessed by CheckM v. 1.0.18 (13), was 98.8%, and there was no contamination, which met the requirements for a high-quality metagenome-assembled genome (MAG) (14). All of the aforementioned tools were used within the KBase software platform (15) and were run with default parameters unless otherwise specified.

The genome was annotated using the NCBI Prokaryotic Genome Annotation Pipeline (PGAP) (16). It contained 24 contigs, which were organized into 18 scaffolds. The sequence total and ungapped lengths were 2,662,706 and 2,662,106 bp, respectively. The GC content was 32.53%, the scaffold *N*_50_ value was 340,628 bp, and the *L*_50_ value was 3. The total number of genes was 2,788, including 2,738 coding sequences (2,699 protein-coding sequences) and 39 pseudogenes. There were 50 RNA genes, including 4 rRNA genes (two 5S, one 16S, and one 23S), 44 tRNA genes, and 2 noncoding RNA genes.

To clarify the taxonomic position of the VT strain, the GTDB-tk (17) was used. The closest related organism was *Methanobacterium* sp. strain SMA-27, which was isolated from frozen soils of Samoilovsky Island (Russia). The VT strain had 95.54% and 65.30% identity to the *Methanobacterium* sp. strain SMA-27 genome (GenBank accession number GCA_000744455) by the average nucleotide identity (ANI) Calculator (https://www.ezbiocloud.net/tools/ani) and DNA-DNA hybridization (DDH) (https://ggdc.dsmz.de), respectively.

*Methanobacterium* sp. strain VT genome information will be useful for further studies of the possible contributions of hydrogen-consuming methanogens from permanently cold habitats to global warming and climate change.

### Data availability.

The raw sequencing reads were deposited in the Sequence Read Archive (SRA) (SRA accession number SRX12179610). The genome sequence of *Methanobacterium* sp. strain VT is available in GenBank under accession number JAIOUQ000000000.1 (BioProject accession number PRJNA760290 and BioSample accession number SAMN21212324), and the 16S rRNA nucleotide sequence was deposited under accession number OK037044.
